# Suppressing the Phase Transformation in Cubic Prussian Blue Analogues via a High-Entropy Strategy for Efficient Zinc-Ion Storage

**DOI:** 10.3390/ma18143409

**Published:** 2025-07-21

**Authors:** Hongwei Huang, Haojun Liu, Yang Wang, Yi Li, Qian Li

**Affiliations:** College of Materials Science and Engineering, and Jiangsu Collaborative Innovation Center for Advanced Inorganic Function Composites, Nanjing Tech University, Nanjing 211816, China; hhw2317452719@163.com (H.H.); liuhaojun1203@163.com (H.L.); liyi19991225@163.com (Y.L.)

**Keywords:** aqueous zinc-ion batteries, cathode, Prussian blue analogs, phase transformation, high-entropy materials

## Abstract

Prussian blue analogs (PBAs) are widely recognized as promising candidates for aqueous zinc-ion batteries (AZIBs) due to their stable three-dimensional framework structure. However, their further development is limited by their low specific capacity and unsatisfactory cycling performance, primarily caused by phase transformation during charge–discharge cycles. Herein, we employed a high-entropy strategy to introduce five different metal elements (Fe, Co, Ni, Mn, and Cu) into the nitrogen–coordinated M_a_ sites of PBAs, forming a high-entropy Prussian blue analog (HEPBA). By leveraging the cocktail effect of the high-entropy strategy, the phase transformation in the HEPBA was significantly suppressed. Consequently, the HEPBA as an AZIB cathode delivered a high reversible specific capacity of 132.1 mAh g^−1^ at 0.1 A g^−1^, and showed exceptional cycling stability with 84.7% capacity retention after 600 cycles at 0.5 A g^−1^. This work provides innovative insights into the rational design of advanced cathode materials for AZIBs.

## 1. Introduction

Currently, lithium-ion batteries are commonly employed in electric vehicles and electronic devices due to their high energy storage and efficiency [1,2]. However, the increasing demand for lithium resources, along with the safety risks associated with flammable organic electrolytes, has highlighted the urgent need for safer and more cost-effective energy storage alternatives [3,4]. Aqueous zinc-ion batteries (AZIBs), are promising candidates for next-generation energy storage owing to their high capacity, low cost, simple fabrication, high safety, and environmental friendliness [5]. In AZIBs, the choice of cathode material significantly influences their electrochemical performance. Thus, developing structurally optimized cathode materials is crucial for advancing commercial AZIBs technologies [6,7,8,9].

To date, a variety of materials have been explored as potential cathodes for AZIBs [10,11,12], including vanadium-based compounds, manganese oxides, polyanionic materials, and Prussian blue analogs (PBAs) [13,14,15]. Among these, PBAs stand out due to their facile synthesis, robust open-framework architecture, and tunable physicochemical and electrochemical properties. PBAs are generally represented by the formula K_x_M_a_[M_b_(CN)_6_]_1−y_□_y_nH_2_O, where M_a_ and M_b_ denote transition metal ions [16,17,18,19], and □ signifies an anionic vacancy within the [M_b_(CN)_6_] framework [20]. The transition metal sites (M_a_ and M_b_) in PBAs exhibit redox activity due to the three-dimensional coordination network formed by metal centers and bridging cyano ligands (–M_a_–N≡C–M_b_–) [21]. This structure provides ample pathways for Zn^2+^ ion transport and creates multiple redox-active sites for enhanced energy storage capacity [22]. For example, Ruo et al. synthesized MnPBA as a cathode material for AZIBs, achieving a capacity of 112 mAh g^−1^ and 67.9% retention after 250 cycles [23]. Xing et al. applied the high-entropy strategy to Mn-based Prussian blue analogs to enhance the cycling stability of AZIBs [24]; however, this approach did not address the issue of possible phase transitions of PBAs during cycling. Despite their high theoretical capacity, PBAs often suffer from structural deformation during cycling due to uncontrolled phase transformation, leading to rapid capacity fading [25].

Herein, we employed a high-entropy strategy to introduce five distinct metal elements into the nitrogen-coordinated M_a_ sites of PBAs, resulting in a high-entropy Prussian blue analog (HEPBA) with a configurational entropy of 1.55 R. The cocktail effect of this high-entropy design effectively suppresses phase transformation, significantly improving both capacity and cycling stability. Consequently, HEPBA cathodes delivered an impressive specific capacity of 132.1 mAh g^−1^ at 0.1 A g^−1^ and showed excellent cycling stability with 84.7% capacity retention after 600 cycles at 0.5 A g^−1^. This work offers new insights into the rational design of cathode materials for advanced AZIBs.

## 2. Experimental Section

### 2.1. Material Preparations

#### 2.1.1. Materials

Potassium ferricyanide (K_3_Fe(CN)_6_, 99.5%), zinc sulfate pentahydrate (ZnSO_4_·5H_2_O, 99.9%), manganese sulfate monohydrate (MnSO_4_·H_2_O, 99.9%), iron(II) chloride tetrahydrate (FeCl_2_·4H_2_O, 98.0%), cobalt nitrate hexahydrate (Co(NO_3_)_2_·6H_2_O, 99.9%), manganese(II) chloride tetrahydrate (MnCl_2_·4H_2_O, 99.0%), nickel nitrate hexahydrate (Ni(NO_3_)_2_·6H_2_O, 99.9%), and copper(II) nitrate trihydrate (Cu(NO_3_)_2_·3H_2_O, 99.0%) were obtained from the Aladdin company, Shanghai, China. Trisodium citrate dihydrate (Na_3_C_6_H_5_O_7_·2H_2_O, analytical reagent grade (AR)) was purchased from the Sinopharm chemical reagent company, Shanghai, China.

#### 2.1.2. Synthesis of High-Entropy Precursor Sample

The HEPBA was prepared through a co-precipitation technique. Initially, 2 mmol of K_3_Fe(CN)_6_ was dissolved in 80 mL of distilled water to form solution A. Separately, solution B was obtained by dissolving 2 mmol of mixed metal salts (FeCl_2_·4H_2_O, Co(NO_3_)_2_·6H_2_O, MnCl_2_·4H_2_O, Ni(NO_3_)_2_·6H_2_O, and Cu(NO_3_)_2_·3H_2_O with each metal salt being 0.4 mmol) and 7 mmol of Na_3_C_6_H_5_O_7_·2H_2_O in 100 mL of deionized water. Subsequently, solution A was gradually added into solution B under continuous stirring for 1 h and allowed to age statically for 48 h. The final product was washed with distilled water and ethanol repeatedly and dried at 60 °C.

#### 2.1.3. Synthesis of Medium-Entropy and Low-Entropy Samples

The medium-entropy Prussian blue analog (MEPBA) and low-entropy Prussian blue analog (LEPBA) were synthesized similarly by adjusting the ratios of metal precursors. The MEPBA used FeCl_2_·4H_2_O, Co(NO_3_)_2_·6H_2_O, MnCl_2_·4H_2_O, and Ni(NO_3_)_2_·6H_2_O with each metal salt being 0.5 mmol. The LEPBA used Co(NO_3_)_2_·6H_2_O and Ni(NO_3_)_2_·6H_2_O with each metal salt being 1 mmol.

### 2.2. Material Characterization

The crystal structure was characterized via X-ray diffraction (XRD, D8 Advance, Bruker Corporation, Billerica, MA, USA) with Kα radiation (λ = 0.1789 nm). The morphology was investigated using scanning electron microscopy (SEM, S-4800, Hitachi High-Technologies Corporation, Tokyo, Japan). Lattice fringes and crystallinity were characterized via transmission electron microscopy (TEM, F200, JEOL Ltd., Tokyo, Japan) with high-resolution imaging (HRTEM) and selected-area electron diffraction (SAED). Elemental distribution was mapped by scanning transmission electron microscopy–energy dispersive X-ray spectroscopy (STEM-EDS, X-MaxN 80 and TIE250, Oxford Instruments, Abingdon, UK). The elemental composition of potassium, iron, copper, cobalt, nickel, and manganese was assessed via inductively coupled plasma mass spectrometry (ICP-MS, 7800, Agilent Technologies Inc., Santa Clara, CA, USA). The content of carbon, hydrogen, and nitrogen was determined by elemental analysis (EA, Vario Micro, Elementar Analysensysteme GmbH, Langenselbold, Germany). The oxidation states of the transition metals were evaluated by X-ray photoelectron spectroscopy (XPS, Nexsa, Thermo Fisher Scientific, Waltham, MA, USA), with the C 1s peak (284.8 eV) as reference. Thermogravimetric analysis (TGA, STA 449, Netzsch-Gerätebau GmbH, Selb, Germany) was performed under a N_2_ atmosphere, with the temperature rising from 25 °C to 400 °C at a 5 °C min^−1^. Fourier transform infrared spectroscopy (FT-IR, TENSOR27, Bruker Corporation, Billerica, MA, USA) was utilized to identify functional group vibrations.

### 2.3. Electrochemical Tests

The cathode slurry (70 wt% active material, 20 wt% Ketjen Black, 10 wt% polyvinylidene fluoride) was coated on the titanium foil and vacuum-dried at 80 °C to achieve a mass loading of 1.5–2.0 mg cm^−2^. CR2032 coin cells were assembled in ambient conditions, with zinc foil as the anode, Whatman glass fiber as the separator, and 2.0 M ZnSO_4_ and 0.2 M MnSO_4_ as the electrolyte. Cyclic voltammetry (CV) and electrochemical impedance spectroscopy (EIS) tests were carried out using an electrochemical workstation (CHI760E, Chenhua, Shanghai, China). Galvanostatic charge–discharge (GCD) and galvanostatic intermittent titration technique (GITT) tests were performed using a battery tester (BTS V00749, Neware, Shenzhen, China).

### 2.4. Calculation Method

The density functional theory (DFT) calculations were carried out by using the Vienna Ab-initio Simulation Package (VASP). The projector augmented wave (PAW) method was employed to expand the wave function [26]. The exchange–correlation energy was described by the Perdew–Burke–Ernzerhof (PBE) functional based on the generalized gradient approximation (GGA) [27]. The cut-off energy for PAW was set as 520 eV through the calculations. The Brillouin zones were sampled with 2 × 2 × 2 k-point meshes. The convergence thresholds were set at 10^−5^ eV for energy and 0.01 eV Å^−1^ for force. The reduction energy (Δ*E*) of PBA after the extraction of K^+^ was calculated as Δ*E* = (*E*_(PBA framework without K)_ + *nE*_(K)_ − *E*_(PBA framework)_)/*n*.

## 3. Results and Discussion

**Figure 1:**
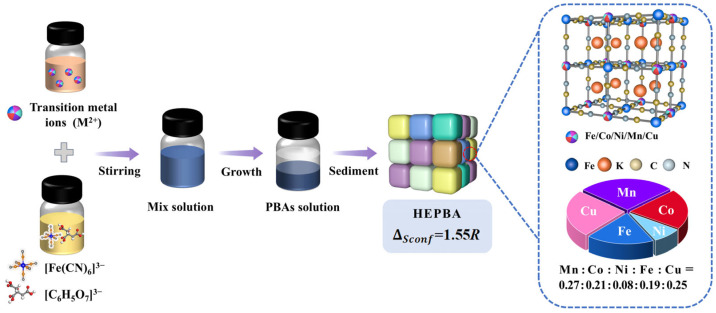
Schematic representation of the synthesis of PBAs by high-entropy strategy.

The synthesis of the HEPBA via a high-entropy strategy is illustrated in Figure 1. Five metal cations (Fe^2+^, Mn^2+^, Co^2+^, Ni^2+^, and Cu^2+^) were introduced using water as the solvent and Na_3_C_6_H_5_O_7_ as the chelating agent. Na_3_C_6_H_5_O_7_ slows down the nucleation and growth rate of materials. Through a simple room-temperature co-precipitation method followed by an aging process, the HEPBA was synthesized. In the HEPBAs, these five cations were expected to distribute at the nitrogen-coordinated sites, forming a three-dimensional framework.

**Figure 2:**
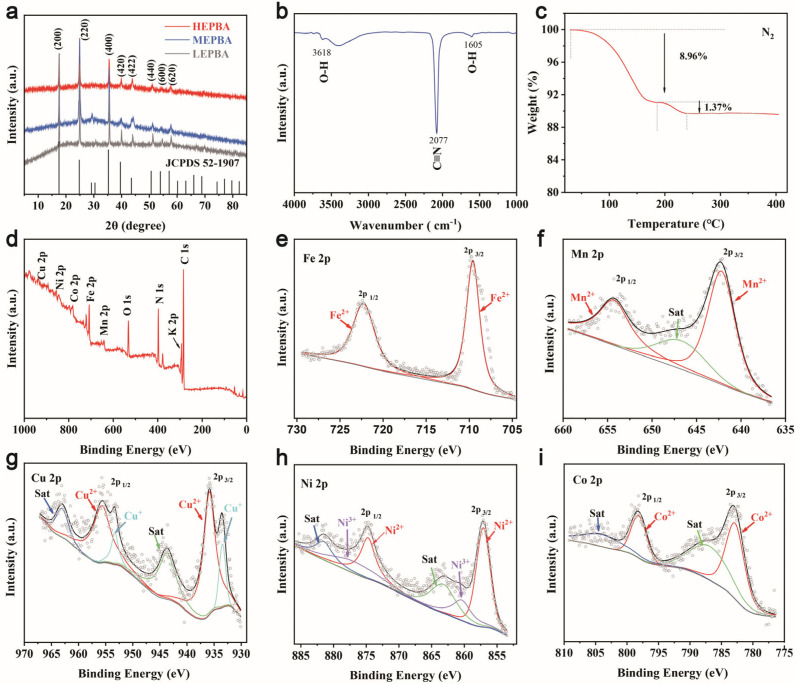
Structural characterization of PBAs. (**a**) Powder XRD patterns of HEPBA, LEPBA, and MEPBA. (**b**) FT-IR curve of HEPBA. (**c**) TGA curve of HEPBA. (**d**) Total XPS spectra of HEPBA, and detailed spectra of (**e**) Fe 2p, (**f**) Mn 2p, (**g**) Cu 2p, (**h**) Ni 2p, and (**i**) Co 2p.

To verify the formation of the PBA structure, XRD analysis was performed. As shown in Figure 2a, the HEPBA, MEPBA, and LEPBA samples all displayed a single-phase cubic crystalline structure, characterized by the Fm-3m space group, matching the JCPDS card No. 52–1907 [28,29]. These XRD results confirmed that incorporating Fe^2+^, Mn^2+^, Co^2+^, Ni^2+^, and Cu^2+^ into the M_a_ sites did not significantly alter the HEPBA framework, preserving its high crystallinity. The FT-IR spectrum of HEPBA is shown in Figure 2b. It presented an absorption peak at 2077 cm^−1^, assigned to the stretching vibration of C≡N bond. Additionally, peaks at 1605 cm^−1^ and 3618 cm^−1^ corresponded to the bending and stretching vibrations of O–H groups, respectively. These characteristic functional groups played a crucial role in maintaining the stability of the PBA framework and providing reliable sites for Zn^2+^ ion intercalation and deintercalation, thereby ensuring favorable electrochemical performance.

The water content in the PBA framework critically influences cell electrochemical performance. As shown in Figure 2c, the TGA curve displayed two evident stages of weight loss: the first stage (below 185 °C) corresponded to the release of physically adsorbed water molecules, while the second stage (above 240 °C) marked the elimination of interstitial and coordinated water molecules, along with the onset of structural decomposition of HEPBA. Lattice vacancies in PBAs can serve as adsorption sites for water molecules, and both coordination adsorption and physical adsorption can affect ion transport and structural stability [30]. Additionally, when water molecules occupy these sites, they hinder the insertion and extraction of zinc ions, leading to a decrease in cycling stability [31]. The relatively low water content in the HEPBA suggests fewer vacancies and a more complete crystal structure, which likely contributes to the formation of a robust structure for achieving long-term electrochemical performance. ICP-MS (Table S1) and EA (Table S2) were employed to evaluate the chemical composition of the HEPBA. The resulting chemical composition was determined to be K_0.68_(Mn_0.27_Co_0.21_Ni_0.08_Fe_0.19_Cu_0.25_)[Fe(CN)_6_]_0.61_□_0.39_·1.11H_2_O (Table S3), with a detailed calculation procedure outlined in Table S4. Based on statistical thermodynamics, the configurational entropy was calculated using the formula

(1) $$\Delta_{\mathrm{Sconf}}=k_{B}\ln\omega_{\mathrm{config}}$$

where $k_{B}$ is the Boltzmann constant and $\omega_{\mathrm{config}}$ is the number of distinct configurational microstates. Since the metal cations in the HEPBA are randomly distributed on the lattice sites of M_a_, the formula can be derived as [32]

(2) $$\Delta_{\mathrm{Sconf}}=-R\sum_{i}^{n}x_{i}\ln x_{i}$$

where *R* denotes the universal gas constant, with *i* and *n* representing the number of transition metals occupying the M_a_ site in M_a_[Fe(CN)_6_]_1-y_□_y_·nH_2_O, and x denotes the mole fraction of the elements occupying the M_a_ site [33]. According to established criteria, materials are classified as low-, medium-, or high-entropy based on their configurational entropy values being less than 1 R, from 1 to 1.5 R, and more than 1.5 R, respectively [34]. The calculations revealed that the HEPBA exhibited a configurational entropy of 1.55 R, thus classifying it as a high-entropy material. Higher configurational entropy values indicate greater structural stability [34], which may lead to improved electrochemical properties by mitigating phase transformation and maintaining structural integrity over extended cycling.

The full XPS spectrum confirmed the existence of Mn, K, Ni, Co, Fe, C, Cu, and N elements (Figure 2d). Detailed fitted curves for each metal element are presented in Figure 2e–i. The Fe 2p spectrum exhibited peaks at 709.7 eV and 722.3 eV, corresponding to Fe 2p_3/2_ and Fe 2p_1/2_, respectively, suggesting that iron was predominantly in the divalent state (Fe^2+^) [35]. The Mn 2p spectrum displayed distinct peaks at 642.3 eV (Mn 2p_3/2_) and 654.5 eV (Mn 2p_1/2_), along with a satellite peak at 647.3 eV, confirming the presence of Mn^2+^ ions [36]. In the Cu 2p region, satellite peaks at 935.9 eV and 955.7 eV indicated the presence of Cu^2+^ ions, while two additional peaks at 933.5 eV and 953.3 eV correspond to Cu^+^ 2p_3/2_ and Cu^+^ 2p_1/2_, respectively, confirming a mixed-valence state for copper within the HEPBA framework [37]. The Ni 2p spectrum revealed two main peaks at 857.2 eV and 874.8 eV, attributed to Ni^2+^ (Ni 2p_3/2_ and Ni 2p_1/2_), with minor peaks at 863.0 eV and 876.8 eV likely associated with Ni^3+^ [38]. Based on these findings, it was inferred that an internal redox reaction occurred during synthesis, leading to the coexistence of Ni^2+^/Ni^3+^ and Cu^+^/Cu^2+^ redox couples. The Co 2p spectrum displayed major peaks at 783.1 eV and 798.2 eV, along with satellite peaks at higher binding energies, characteristic of Co^2+^ [36].

**Figure 3:**
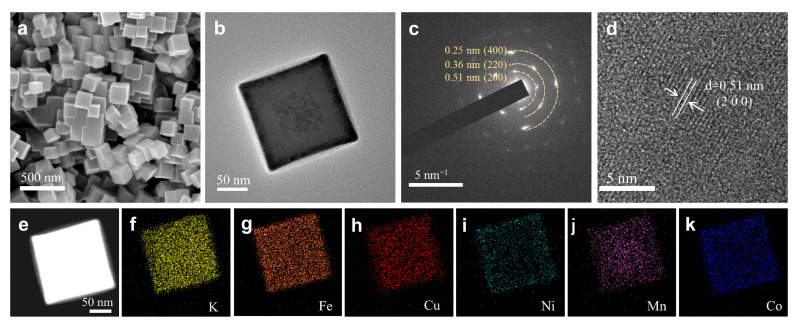
Structural and morphological characterization of HEPBA. (**a**) SEM, (**b**) TEM, (**c**) SAED, and (**d**) HRTEM images of HEPBA. (**e**) STEM image of HEPBA and (**f**–**k**) its corresponding elemental mapping results.

The morphology and microstructure of the HEPBA were revealed by SEM and TEM. As displayed in Figure 3a,b, the HEPBA nanoparticles exhibit a well-defined cubic shape with uniform distribution. TEM images revealed a particle size of ~200 nm for HEPBA. Similarly, MEPBA and LEPBA also exhibit a cubic morphology with a particle size of approximately 200 nm (Figure S1), and EDS demonstrated the uniform elemental distribution within these materials (Figures S2 and S3). Notably, the morphologies of the MEPBA and LEPBA exhibited some degree of agglomeration in contrast to the homogeneous structure of the HEPBA. This suggested that the introduction of entropy in the HEPBA enhanced the homogeneity of the PBAs, potentially contributing to their high specific capacity. The SAED pattern in Figure 3c displayed distinct diffraction rings corresponding to the (200), (220), and (400) lattice planes, confirming the polycrystalline characteristics of the HEPBA. As shown in Figure 3d, the HRTEM revealed clear lattice fringes with an interplanar spacing of 0.51 nm, consistent with the (200) plane separation distance determined through SAED. These findings further confirmed that the synthesized material was the HEPBA with the Fm-3m space group. In addition, STEM imaging (Figure 3e) and corresponding elemental mapping (Figure 3f–k) further confirmed the uniform distribution of Mn, Ni, Fe, Co, and Cu in the HEPBA.

**Figure 4:**
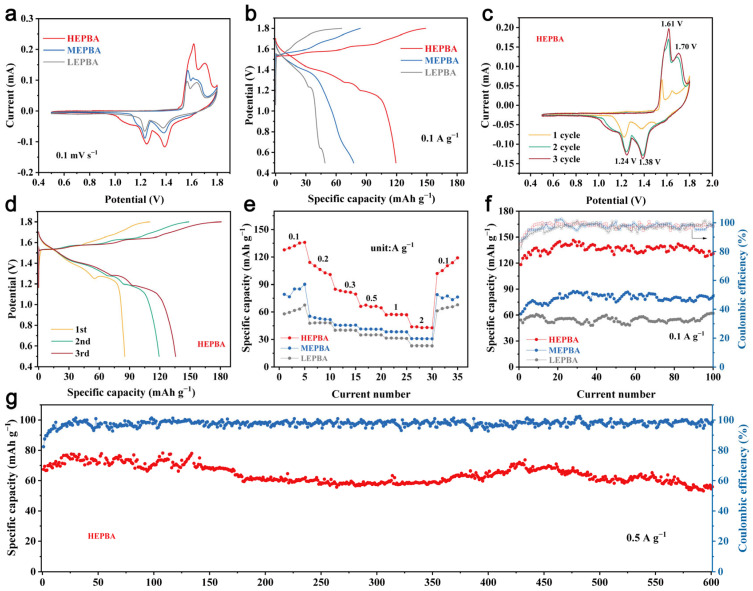
Electrochemical performance of the HEPBA, MEPBA, and LEPBA cathodes in AZIBs. (**a**) CV curves of electrode materials at 0.1 mV s^−1^. (**b**) GCD curves at 0.1 A g^−1^. (**c**) Initial CV curves of the HEPBA for the first three cycles at 0.1 mV s^−1^. (**d**) The GCD curves of the HEPBA for the first three cycles at 0.1 A g^−1^. (**e**) Multiplication capabilities from 0.1 to 2 A g^−1^. (**f**) The cycling performance of the HEPBA, MEPBA, and LEPBA at 0.1 A g^−1^. (**g**) The long-term cycling performance of the HEPBA at 0.5 A g^−1^.

The electrochemical performance of the LEPBA, MEPBA, and HEPBA was comprehensively investigated (Figure 4). As shown in Figure 4a, the area under the CV curves increased with configurational entropy, indicating that higher entropy enhanced their electrochemical performance. In Figure 4b, GCD curves were presented at 0.1 A g^−1^. Each electrode material exhibited two distinct voltage plateaus corresponding to the redox reactions involving N-coordinated M_a_ and C-coordinated Fe. Notably, the HEPBA showed a longer discharge plateau than the MEPBA and LEPBA, indicating enhanced charge storage capabilities. The first three CV cycles of the HEPBA at 0.1 mV s^−1^ are illustrated in Figure 4c. The material stabilized after the second cycle, with the CV curves of the subsequent cycles overlapping closely, demonstrating excellent reversibility. Two redox couples were observed at 1.70/1.38 V (N-coordinated M_a_^2+/3+^) and 1.61/1.24 V (C-coordinated low-spin Fe^2+/3+^), indicating distinct metal centers with different coordination environments. Moreover, a broader peak at 1.1 V suggested a solid–solution reaction during cycling, which was similar to previous reports [39]. This was further corroborated by the GCD profile in Figure 4d, where the high-potential plateau remained stable while the low-potential plateau extended over time.

The rate performance test in Figure 4e revealed that the HEPBA outperformed the MEPBA and LEPBA across various current densities. Within the current density range of 0.1 to 2 A g^−1^, the HEPBA maintained capacities of 132.1, 106.8, 82.3, 66.0, 57.1, 43.2 mAh g^−1^, respectively (Figure S4). After high-rate testing, the HEPBA recovered a discharge capacity of 110.1 mAh g^−1^ at 0.1 A g^−1^, highlighting its outstanding rate capability and structural reversibility.

The cycling performance at 0.1 A g^−1^ (Figure 4f) showed that all samples initially increased in capacity before stabilizing after approximately 100 cycles, indicative of a typical electrochemical activation process. Even at higher currents like 0.5 A g^−1^ (Figure 4g), the HEPBA demonstrated remarkable stability, starting with an initial specific capacity of 67.4 mAh g^−1^ and increasing to 76.8 mAh g^−1^ after 20 cycles. In comparison, the MEPBA and LEPBA delivered only 45.9 mAh g^−1^ and 37.9 mAh g^−1^, respectively. Regarding long-term cycling performance, the HEPBA retained 84.7% of its capacity after 600 cycles at 0.5 A g^−1^, whereas the MEPBA and LEPBA showed rapid capacity degradation after only 400 cycles (Figures S5 and S6). These findings underscored the superior charge/discharge stability of the HEPBA, and a comparative analysis with other PBA-based cathodes (Table S5) further highlighted the competitive advantage of the HEPBA, confirming that the high-entropy strategy significantly improved both discharge capacity and long-term cycling performance.

**Figure 5:**
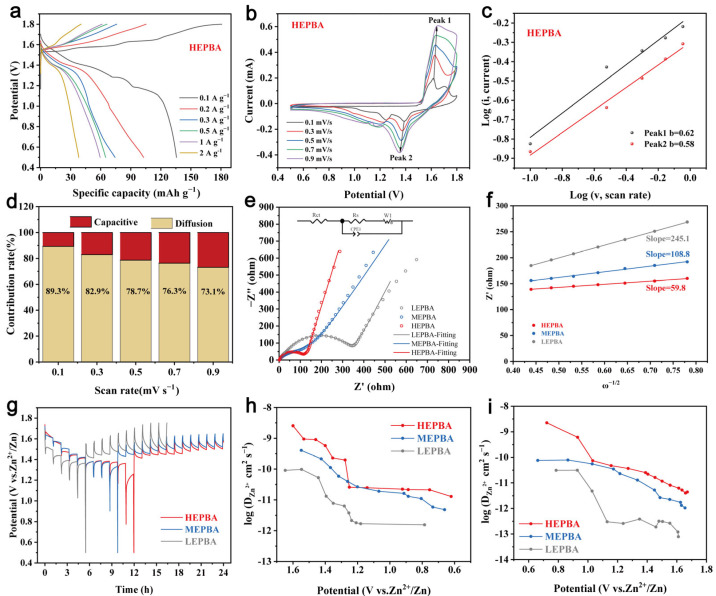
(**a**) GCD plots of HEPBA at different current densities. (**b**) CV curves of HEPBA at different sweep speeds. (**c**) Corresponding b-values and (**d**) pseudocapacitance calculations of HEPBA. (**e**) EIS of HEPBA, MEPBA, and LEPBA electrodes. (**f**) Linear fitting of Z′ vs. w^−1/2^. (**g**) GITT curves and Zn^2+^ diffusion coefficient curves for (**h**) discharge and (**i**) charge processes.

To elucidate the influence of configurational entropy on electrochemical performance, GCD profiles of the HEPBA were recorded at various current densities (Figure 5a). At 0.1 A g^−1^, the material exhibited two distinct discharge plateaus. However, as the current density increased, these two plateaus converged into one single plateau. This likely occurred because the material could not facilitate timely Zn^2+^ intercalation and deintercalation, leading to the overlap of the two plateaus. Figure 5b presents CV curves at different scan rates. As the scan rate increased, the redox peaks gradually merged into a single pair, indicating that the rapid scan rate outpaced the intrinsic reaction kinetics. This phenomenon was also observed in the MEPBA and LEPBA (Figures S7 and S8), and is consistent with the GCD curve observations. The electrochemical kinetics of the HEPBA electrode were further evaluated by investigating the relationship between peak current and scan rate. Typically, the peak current (*i*) could be represented as [40]

(3) $$i=av^{b}$$

(4) logi=blogv+bloga

where *a* is a constant and *b* (0 ≤ *b* ≤ 1) serves as a parameter that aids in identifying the charge storage mechanism of the electrode material. For the HEPBA, the calculated *b*-values for Peak 1 and Peak 2 were 0.62 and 0.58, respectively, both close to 0.5, implying that the charge storage was predominantly governed by diffusion-controlled mechanisms (Figure 5c).

This analysis quantitatively differentiated the contributions of capacitive and diffusive behaviors to the total capacity, which were represented as [41]

(5) $$i=k_{1}v+k_{2}v^{1/2}$$

(6) $$i/v^{1/2}=k_{1}v^{1/2}+k_{2}$$

where *i*, $k_{1}v$, and $k_{2}v^{1/2}$ denote the total current, the contribution from capacitive behavior, and the contribution from diffusive behavior, respectively. It was observed that the diffusion-controlled mechanism dominated the electrochemical charge storage process in the HEPBA electrode (Figure 5d), with 73.1% of the total current attributed to diffusion control, even at a high scan rate of 0.9 mV s^−1^. This finding aligned with the results reported by Niragatti et al. [42].

EIS was further employed to probe the Zn^2+^ ion intercalation/deintercalation kinetics (Figure 5e). Within the equivalent circuit model, *R_s_* represented the resistance of the electrolyte, *R_ct_* denoted the charge transfer resistance occurring at the electrode-electrolyte interface, and CPE was the constant phase element [43]. Notably, the HEPBA electrode exhibited a lower *R_ct_* value (87.0 Ω) compared to the MEPBA (105.4 Ω) and LEPBA (392.1 Ω) (Table S6), implying that the multi-elemental configuration facilitated Zn^2+^ diffusion and accelerated interfacial charge transfer, thereby enhancing cycling stability. Furthermore, the increase in configurational entropy aided in reducing the resistance between the electrolyte and the electrode material, particularly the Warburg impedance. As illustrated in Figure 5f, the slope corresponded to the Warburg impedance factor *σ*, with values of *σ* for the HEPBA, MEPBA, and LEPBA being 59.8, 108.8, and 245.1, respectively. The lower Warburg impedance provided a theoretical basis for the superior electrochemical performance of the HEPBA.

The Zn^2+^ diffusion coefficients for the three electrode materials were determined via the GITT

(7) $$D_{{Zn}^{2+}}=(\frac{4}{\pi \tau }){\left(\frac{n_{m}V_{m}}{A}\right)}^{2}{\left(\frac{∆E_{s}}{∆E_{t}}\right)}^{2}$$

Here, *τ*, $n_{m}$, $V_{m}$, A, $∆E_{s}$, and $∆E_{t}$ are the relaxation times related to the number of moles, molar mass, molar volume, contact area, and voltage differences during a single pulse [44]. As illustrated in Figure 5g–i, the GITT test revealed that the diffusion coefficient for the HEPBA ranges from 10^−8^ to 10^−11^ cm^2^ s^−1^ during the discharge process, significantly higher than those for the MEPBA (10^−9^ to 10^−12^ cm^2^ s^−1^) and LEPBA (10^−10^ to 10^−12^ cm^2^ s^−1^). Additionally, the HEPBA exhibited the smallest fluctuation in diffusion coefficients during the charge process, suggesting more consistent and efficient Zn^2+^ ion transport. These results demonstrated that introducing high configurational entropy significantly enhanced Zn^2+^ diffusion kinetics in the HEPBA, along with excellent charge transfer kinetics due to the synergistic effects of diverse elements. Such improvements lead to a substantial reduction in Warburg impedance, thereby enabling accelerated ion transport and diffusion. This kinetic advantage played a pivotal role in achieving the superior electrochemical performance observed in the HEPBA.

**Figure 6:**
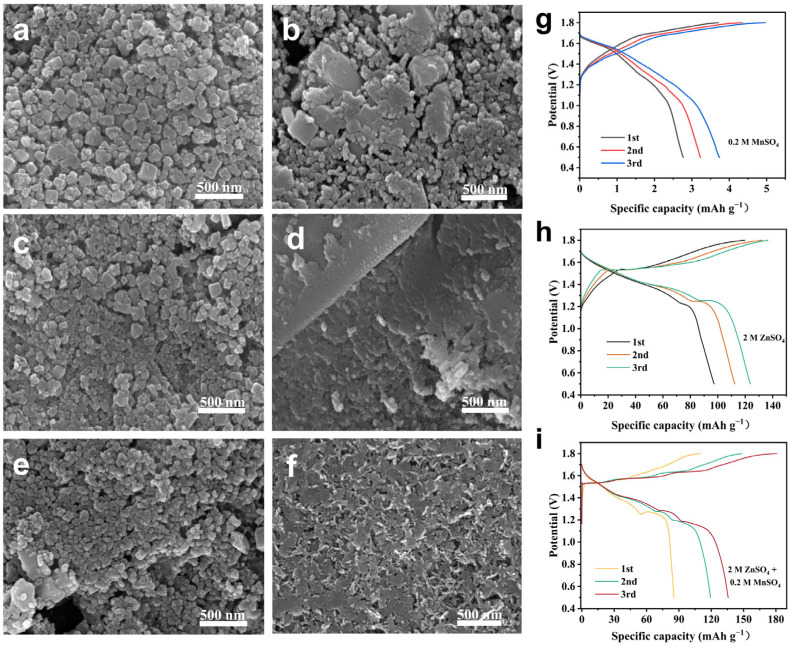
SEM images of (**a**,**b**) the HEPBA, (**c**,**d**) MEPBA, and (**e**,**f**) LEPBA electrodes after the 10th and 100th cycles at a current density of 0.5 A g^−1^. GCD curves of the HEPBA under different electrolytes. (**g**) 0.2 M MnSO_4_ and 2 M ZnSO_4_. (**h**) 2 M ZnSO_4_. (**i**) 0.2 M MnSO_4_.

To investigate the morphological evolution of the three materials during the charge and discharge process, SEM tests were conducted at various cycling stages. The HEPBA electrode exhibits minimal morphological changes after 100 cycles compared to its state at the 10th cycle (Figure 6a,b), with its cubic morphology remaining clearly discernible. In stark contrast, the MEPBA and LEPBA electrodes undergo significant structural degradation after 100 cycles relative to their condition at the 10th cycle, likely due to volume changes during cycling (Figure 6c−f). This morphological stability explains the superior cycling performance of HEPBA, which could withstand 600 cycles at 0.5 A g^−1^ while the MEPBA and LEPBA only maintain a cycle life up to 400 cycles. After 600 cycles (Figure S9), the HEPBA undergoes significant morphological changes. The initially granular structure had almost disappeared, accompanied by the formation of cracks, leaving only a few small particles scattered on the surface. Such severe degradation is likely the main factor contributing to the observed decline in electrochemical performance. In addition, the contribution of Mn^2+^ ions in the electrolyte to capacity was evaluated (Figure 6g−i), the HEPBA cathode in pure MnSO_4_ electrolyte delivered negligible discharge capacity (<5 mAh g^−1^) (Figure 6g), Figure 6h shows that when cycling to the third cycle, the maximum discharge capacity is 123.6 mAh g^−1^, and whereas it exhibited 135.6 mAh g^−1^ (third cycle) in the Zn^2+^-containing electrolyte (2 M ZnSO_4_ and 0.2 M MnSO_4_). The revealing negligible involvement of manganese species, with the high capacity arises from Zn^2+^ ion storage rather than Mn deposition.

**Figure 7:**
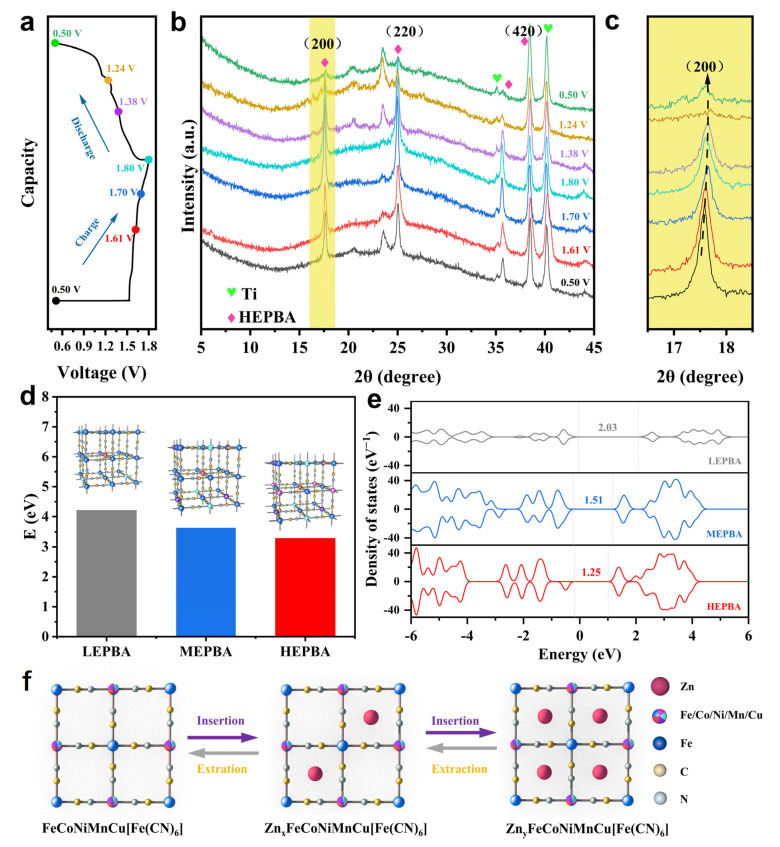
(**a**) The charge/discharge curve of the HEPBA at 0.1 A g^−1^. (**b**) The corresponding ex situ XRD pattern of the HEPBA at various voltage states, and (**c**) the enlarged view of the corresponding (200) plane. (**d**) The reduction energy and optimized geometries of the LEPBA, MEPBA, and HEPBA after ion removal. (**e**) The density of states curves of the LEPBA, MEPBA, and HEPBA. (**f**) A schematic diagram of the Zn^2+^ ion storage mechanism of the HEPBA.

To elucidate the structural evolution of the HEPBA during charging and discharging, ex situ XRD measurements were conducted within the 0.5–1.8 V voltage window (Figure 7a–c). The cubic phase diffraction peaks, particularly the (200), (220), and (420) planes, exhibited reversible shifts during charging and discharging, which are associated with the lattice deformation induced by Zn^2+^ deintercalation/intercalation. The peaks at 20.6° and 23.5° corresponded to the Zn-intercalated HEPBA (ZnHEPBA) [45,46], with their intensity variations correlating to Zn^2+^ intercalation and deintercalation. Specifically, peak intensities decreased during Zn^2+^ extraction and increased upon Zn^2+^ reinsertion. These observations demonstrated the structural reversibility of the HEPBA.

To further investigate the high electrochemical performance enabled by the high-entropy effect, DFT calculations were conducted. As shown in Figure 7d, the calculated reduction energy of the HEPBA after ion removal was 3.28 eV, which was lower than that of the LEPBA (4.21 eV) and MEPBA (3.63 eV). This result indicates that the HEPBA exhibited superior structural stability. The corresponding geometric configurations revealed that the HEPBA undergoes minimal lattice distortion upon ion removal, further confirming the stabilizing effect of the high-entropy design. Figure 7e showed the density of states (DOS) curves for the three materials. The HEPBA exhibited the smallest band gap (1.25 eV) compared to the LEPBA and MEPBA, indicating its improved electronic conductivity. This enhanced conductivity provided theoretical support for the fast reaction kinetics and large capacity observed in the HEPBA.

The Zn^2+^ storage mechanism within the three-dimensional open framework of the HEPBA is schematically illustrated in Figure 7f. During discharging, Zn^2+^ ions were inserted into the open structure of the HEPBA and were subsequently extracted during charging, enabling a stable and reversible electrochemical reaction. This remarkable structural stability stemmed from the random arrangement of five distinct transition metal ions in the crystal lattice, resulting in a diverse set of M_a_^2+/3+^–N coordination bonds. These ions, characterized by differences in charge, ionic radius, and electronic configuration, collectively contributed to forming an intricate and resilient framework with enhanced configurational entropy. The structural complexity facilitates the coordinated atomic motion of these metal ions during Zn^2+^ intercalation/deintercalation, effectively suppressing phase transitions, which is crucial to ensuring long-term cyclic stability.

## 4. Conclusions

This work introduced a high-entropy strategy to suppress phase transformation in cubic PBAs for efficient zinc-ion storage. By incorporating five distinct transition metals (Fe^2+^, Mn^2+^, Co^2+^, Ni^2+^, and Cu^2+^) at nitrogen-coordinated M_a_ sites, the HEPBA achieved a configurational entropy of 1.55 R, forming a robust framework with diverse M_a_^2+/3+^–N coordination bonds. Experimental analysis and DFT calculations demonstrated that the HEPBA possessed superior thermodynamic stability (3.28 eV reduction energy) and minimal lattice distortion compared to the MEPBA and LEPBA, effectively suppressing phase transformation during cycling. The synergistic interplay of multiple metal ions reduced the bandgap to 1.25 eV, enhancing electronic conductivity and reaction kinetics. As a cathode for AZIBs, the HEPBA delivered a high reversible capacity of 132.1 mAh g^−1^ at 0.1 A g^−1^, and showed excellent cycling stability with 84.7% capacity retention after 600 cycles at 0.5 A g^−1^, outperforming previous PBA-based electrodes [23,36,47,48]. This work establishes a universal principle for entropy-stabilized electrodes, where compositional disorder stabilizes phase stability and unlocks synergistic metal effects, providing novel insights for advanced AZIB cathodes and scalability for next-generation multivalent aqueous battery systems.

## Data Availability

The original contributions presented in this study are included in the article/Supplementary Material. Further inquiries can be directed to the corresponding authors.

## Supplementary Materials

The following supporting information can be downloaded at: https://www.mdpi.com/article/10.3390/ma18143409/s1, Figure S1. SEM images of (a) LEPBA, (b) MEPBA and (c) HEPBA. Figure S2. (a) SEM image of MEPBA and (b-i) corresponding EDS mapping of different elements. Figure S3. (a) SEM image of LEPBA and (b-h) corresponding EDS mapping of different. Figure S4. Discharge specific capacities of HEPBA at different current densities. (Average of 5 tests for each current density), Figure S5. Long-term cycling performance of MEPBA at 0.5 A g^−1^. Figure S6. Long-term cycling performance of LEPBA at 0.5 A g^−1^. Figure S7. CV curves of MEPBA at different sweep speeds. Figure S8. CV curves of LEPBA at different sweep speeds. Figure S9. SEM images of HEPBA electrodes after the 600th cycles at a current density of 0.5 A g^−1^. Table S1. ICP-MS of HEPBA. Table S2. EA of HEPBA. Table S3. Chemical formula of HEPBA. Table S4. Calculation of the formula: Table S5. Comparison of electrochemical performances of HEPBA with some previous reported PBA-based cathodes in aqueous zinc-ion batteries. Table S6. Fitted parameters of equivalent circuits for Nyquist plots. References [23,36,47,48,49,50,51] are cited in the Supplementary Materials.
