# Supplementary material for: Suppressing the Phase Transformation in Cubic Prussian Blue Analogues via a High-Entropy Strategy for Efficient Zinc-Ion Storage
Source: Materials (Basel). 2025 Jul 21;18(14):3409. doi: 10.3390/ma18143409 (PMC12297942; doi:10.3390/ma18143409)
Supplement: Supplementary file 1 [file materials-18-03409-s001.zip › materials-3731847-supplementary.pdf]

Supporting Information

Suppressing the phase transformation in cubic Prussian blue analogues via a high-entropy strategy for efficient zinc ion storage

Hongwei Huang, Haojun Liu, Yang Wang *, Yi Li and Qian Li *

College of Materials Science and Engineering, and Jiangsu Collaborative Innovation Center for Advanced Inorganic Function Composites, Nanjing Tech University, Nanjing 211816, China;
hhw2317452719@163.com (H.H.); liuhaojun1203@163.com (H.L.); liyi19991225@163.com (Y.L.)
* Correspondence: wangyang890916@126.com (Y.W.); liqian1004@njtech.edu.cn (Q.L.)

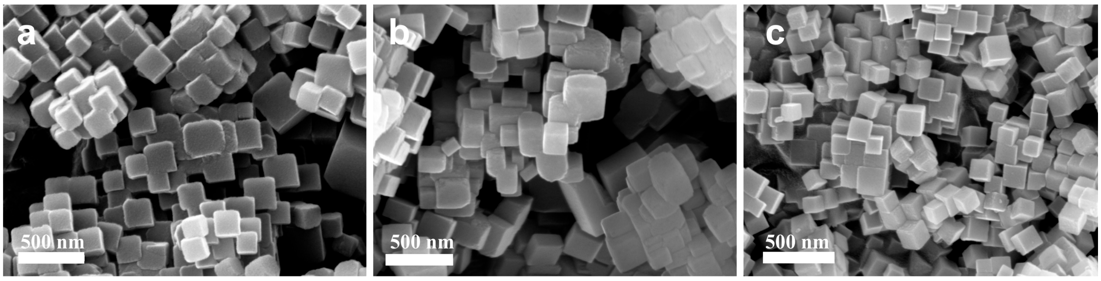

Figure S1. SEM images of (a) LEPBA, (b) MEPBA and (c) HEPBA.

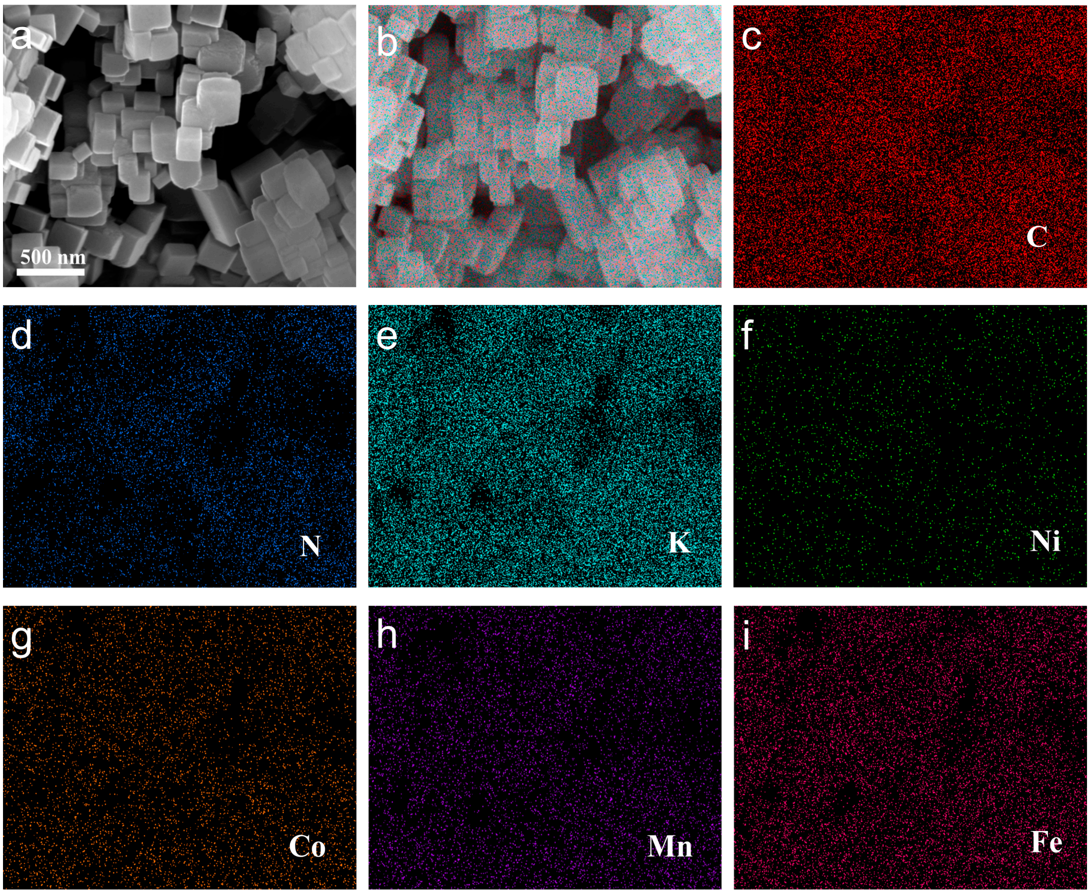

Figure S2. (a) SEM image of MEPBA and (b-i) corresponding EDS mapping of different elements.

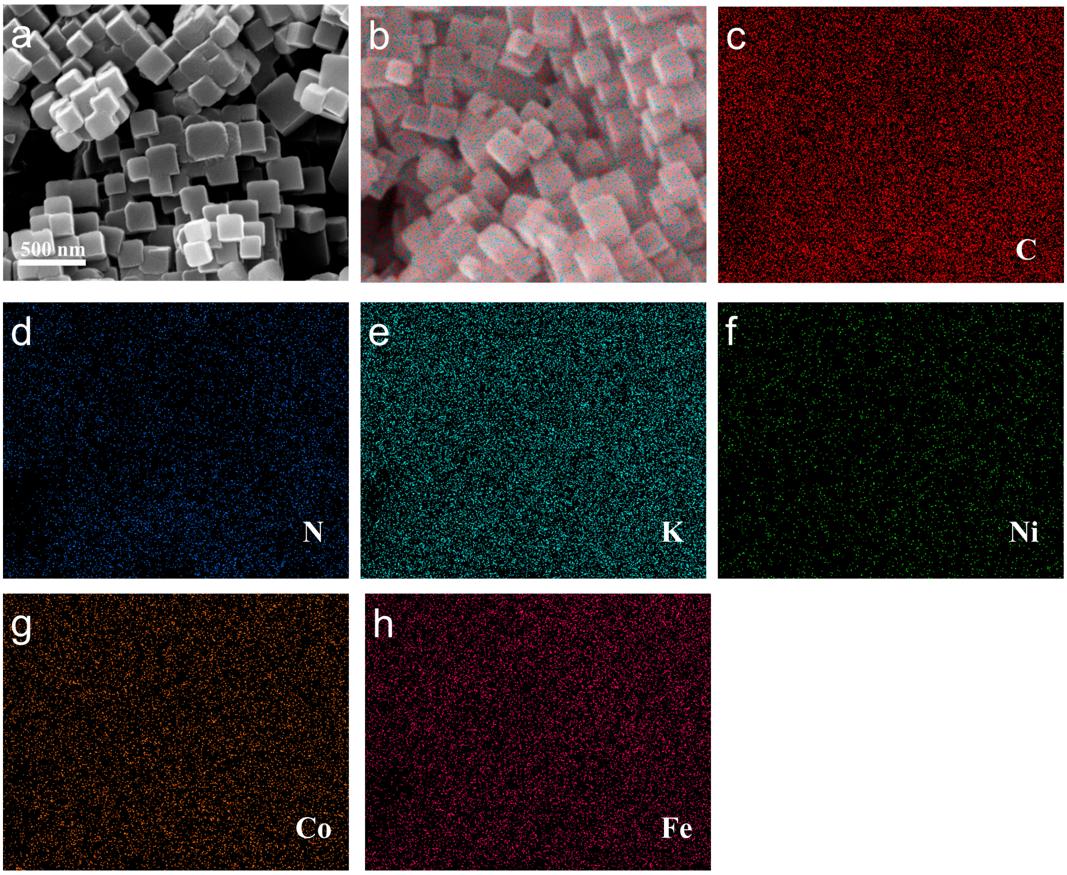

Figure S3. (a) SEM image of LEPBA and (b-h) corresponding EDS mapping of different elements.

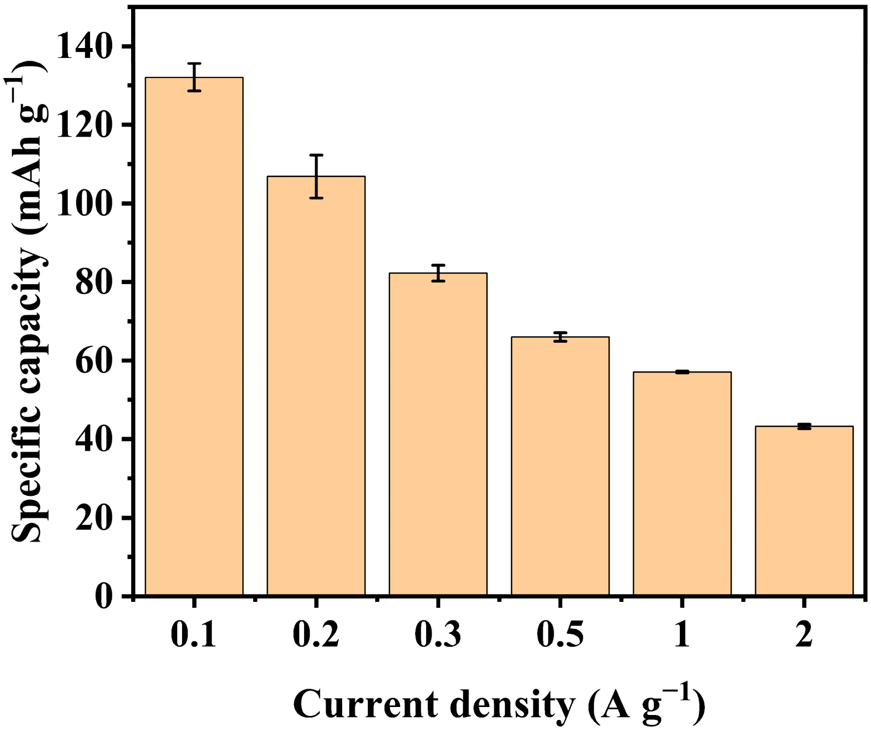

Figure S4. Discharge specific capacities of HEPBA at different current densities. (Average of 5 tests for each current density.)

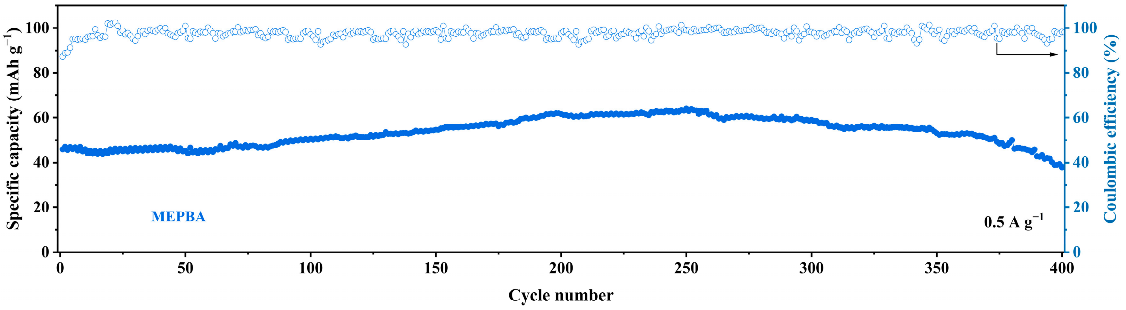

Figure S5. Long-term cycling performance of MEPBA at 0.5 A g^{-1}.

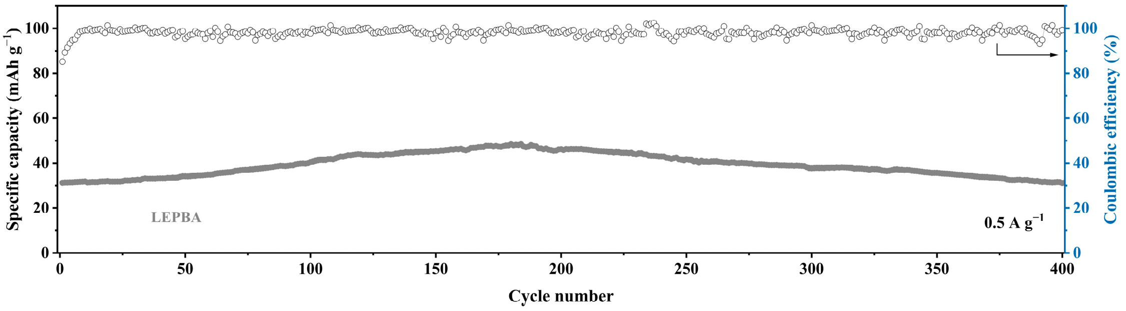

Figure S6. Long-term cycling performance of LEPBA at 0.5 A g^{-1}.

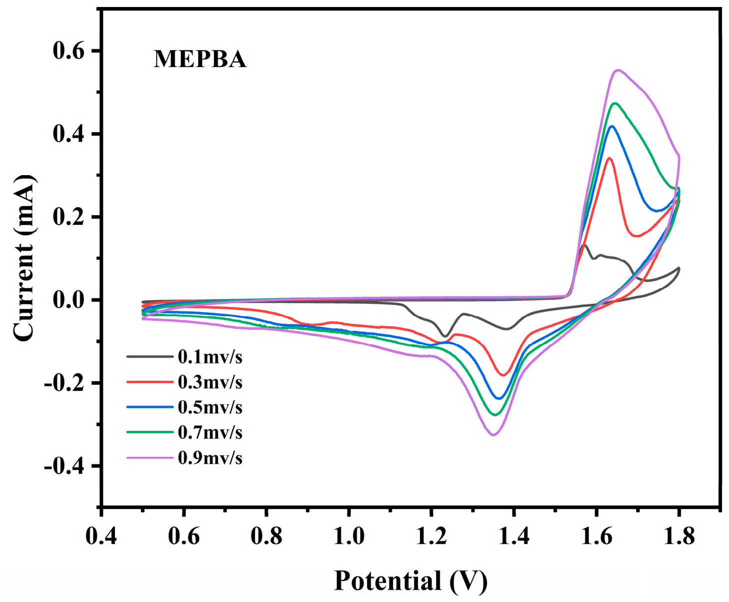

Figure S7. CV curves of MEPBA at different sweep speeds.

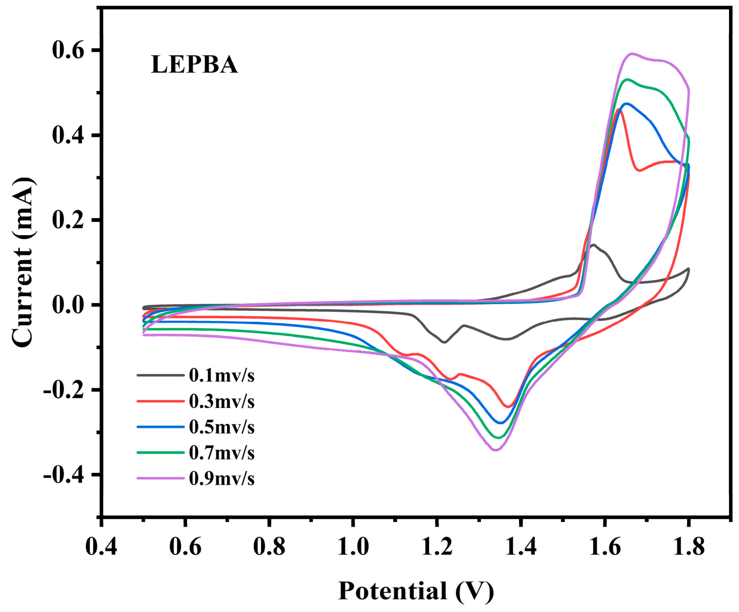

Figure S8. CV curves of LEPBA at different sweep speeds.

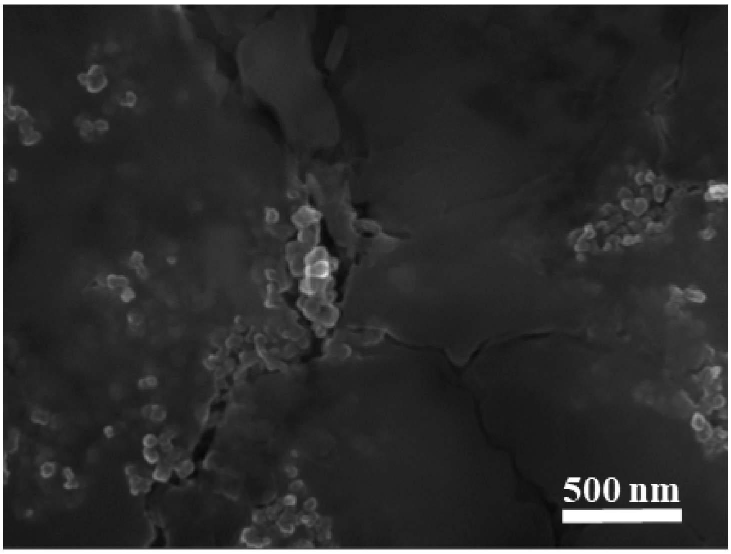

Figure S9. SEM images of HEPBA electrodes after the 600th cycles at a current density of 0.5 A g^{-1}.

Table S1. ICP-MS of HEPBA.

Sample	K(wt%)	Fe(wt%)	Mn(wt%)	Co(wt%)	Ni(wt%)	Cu(wt%)
HEPBA	11.18	18.83	6.37	5.24	2.03	6.62

Table S2. EA of HEPBA.

Sample	C(wt%)	N(wt%)	H(wt%)
HEPBA	19.28	21.68	0.95

Table S3. Chemical formula of HEPBA.

Sample	Chemical formula
HEPBA	$K_{0.68}(Mn_{0.27}Co_{0.21}Ni_{0.08}Fe_{0.19}Cu_{0.25})[Fe(CN)_6]_{0.61}\square_{0.39}\cdot 1.11H_2O$

Table S4. Calculation of the formula:

$$n_{(cn)_6} = \frac{N\ wt\%}{MN * 6}$$

$$\text{Metal\%} = K\% + Fe\% + Mn\% + Co\% + Ni\% + Cu\%$$

$$n_{TMHS} = \frac{TM\%/Metal\% * (1 - Cwt\% - Nwt\% - 9 * Hwt\%)}{M_{TM}}$$

$$n_{Fe_{total}} = \frac{Fe\%/Metal\% * (1 - Cwt\% - Nwt\% - 9 * Hwt\%)}{M_{Fe}}$$

$$n_K = \frac{K\%/Metal\% * (1 - Cwt\% - Nwt\% - 9 * Hwt\%)}{M_K}$$

$$n_{Fe_{HS}} = n_{Fe_{total}} - n_{(cn)_6}$$

$$n_{H_2O} = \frac{Hwt\%}{2 * M_H}$$

$n_{Fe_{HS}}$ stands for the high-spin Fe.

All the calculated results are normalized by $\sum TM^{HS}$

Table S5. Comparison of electrochemical performances of HEPBA with some previous reported PBA-based cathodes in aqueous zinc-ion batteries.

Electrodes	Inserted ions	Electrolytes	Capacity(mAh g^{-1}@A g^{-1})	Cycling life(cycles, retention@A g^{-1})	Ref.
HEPBA	Zn^{2+}	2 M $ZnSO_4$ + 0.2 M $MnSO_4$	132.1@0.1	600(84.7%)@0.5	This work
TiPBA	Zn^{2+}	1 M $ZnSO_4$ + 0.1 M Na_2SO_4	105.8@0.05	300(28.5%)@0.3	[1]
MnCoHCF	Zn^{2+}	1 M $Zn(CF_3SO_3)_2$	128.7@0.1	200(51.2%)@0.1	[2]
MnPBA	Zn^{2+}	1 M $ZnSO_4$	120.5@0.1	1000(65%)@0.5	[3]
CoMnPBA/PANI	Zn^{2+}	2 M $Zn(CF_3SO_3)_2$	158.3@0.1	1000(66.2%)@0.1	[4]
CoNiPBA	Zn^{2+}	3 M $Zn(CF_3SO_3)_2$	124.9@0.05	1000(81.8%)@3	[5]
MnPBA	Zn^{2+}	0.2 M $Zn(CF_3SO_3)_2$ in acetonitrile	112@0.05	250(67.9%)@0.1	[6]
ZnVHCF@rGO	Zn^{2+}	3 M $Zn(CF_3SO_3)_2$	126.7@0.2	300(46.9%)@5	[7]

Table S6. Fitted parameters of equivalent circuits for Nyquist plots.

Samples	Rs/Ohm	Rct/Ohm	$\omega^{-1/2}$
LEPBA	3.3	392.1	245.1
MEPBA	1.6	105.4	108.8
HEPBA	2.1	87.0	59.8

References

[1] D.P. Zhang, D. Wang, X.L. Mao, Z.Y. Zhou, J.H. Zhang, T.F. Ma, Y.H. Zhang, T.J. Yan, Titanium hexacyanoferrate/carbon nanotube composites as the cathode material for aqueous sodium/zinc ion batteries, J. Power Sources. 613 (2024) 234929. https://doi.org/10.1016/j.jpowsour.2024.234929.

[2] J. Zhou, Y. Wang, Z. Wang, Q. Zhang, Z. Hu, Y. Feng, Y. Li, K. Chen, N. Qin, J. Liu, L. Mi, Co/Mn ratio-regulated hexacyanoferrates as a long-life and high-rate cathode for aqueous Zn-ion batteries, J. Alloys Compd. 976 (2024) 173158. https://doi.org/10.1016/j.jallcom.2023.173158.

[3] L. Luo, Y. Liu, Z.X. Shen, Z.R. Wen, S. Chen, G. Hong, High-voltage and stable manganese hexacyanoferrate/zinc batteries using gel electrolytes, ACS Appl. Mater. Interfaces. 15(24) (2023) 29032-29041. https://doi.org/10.1021/acsami.3c00905.

[4] S. Liu, Z.W. Sun, B.G. Li, X.J. Liu, C.G. Xue, Innovative cobalt manganese-based prussian blue analogue/polyaniline cathode materials with double layered hollow nanocube structure for high performance aqueous zinc ion battery, J. Energy Storage. 111 (2025) 115310. https://doi.org/10.1016/j.est.2025.115310.

[5] J.Y. Cao, Y.T. Xue, Z.Y. Ji, J.R. Pu, X.P. Shen, L.R. Kong, A.H. Yuan, CoNi hexacyanoferrate nanoparticles anchored on carbon nanotubes as superior cathode materials for rechargeable aqueous zinc-ion batteries, J. Energy Storage. 86 (2024) 111413. https://doi.org/10.1016/j.est.2024.111413.

[6] H. Ruo, L. Chen, J.L. Huang, C.H. Lv, J.J. Bai, S.D. Xu, J.Q. Chen, D. Zhang, H.M. Yang, Constructing low-cost stable zinc-ion batteries with sodium-rich monoclinic manganese hexacyanoferrate cathode, Surf. Interfaces. 51 (2024) 104594. https://doi.org/10.1016/j.surfin.2024.104594.

[7] B.B. Hu, D.S. Li, M.X. Li, J.Y. Jiang, Y. Zou, Y. Deng, Z.D. Zhou, H. Pu, G.Q. Ma, Z. Li, Conductive network enhanced self-assembled diphasic prussian blue analogs for aqueous zinc-ion batteries, J. Mater. Chem. C. 13(13) (2025) 6736-6744.

https://doi.org/10.1039/d4tc05159a.
